# L-methionine and the L-type Ca^2+^ channel agonist BAY K 8644 collaboratively contribute to the reduction of depressive-like behavior in mice

**DOI:** 10.3389/fncir.2024.1435507

**Published:** 2024-08-29

**Authors:** Ershu He, Ruixue Ma, Shanglan Qu, Xiaoye Zheng, Xin Peng, Jieyu Ji, Wenhao Ma, Xueyan Zhang, Ying Li, Hanwei Li, Yanjiao Li, Lijuan Li, Zhiting Gong

**Affiliations:** ^1^School of Medicine, Dali University, Dali, China; ^2^Faculty of Health and Medical Sciences, School of Pharmacy, Taylor’s University, Subang Jaya, Malaysia

**Keywords:** Cacna1C, Dnmt3a, depression, venlafaxine, L-methionine

## Abstract

The L-type Ca^2+^ channel (LTCC, also known as Cav1,2) is involved in the regulation of key neuronal functions, such as dendritic information integration, cell survival, and neuronal gene expression. Clinical studies have shown an association between L-type calcium channels and the onset of depression, although the precise mechanisms remain unclear. The development of depression results from a combination of environmental and genetic factors. DNA methylation, a significant epigenetic modification, plays a regulatory role in the pathogenesis of psychiatric disorders such as posttraumatic stress disorder (PTSD), depression, and autism. In our study, we observed reduced Dnmt3a expression levels in the hippocampal DG region of mice with LPS-induced depression compared to control mice. The antidepressant Venlafaxine was able to increase Dnmt3a expression levels. Conversely, Bay K 8644, an agonist of the L-type Ca^2+^ channel, partially ameliorated depression-like behaviors but did not elevate Dnmt3a expression levels. Furthermore, when we manipulated DNA methylation levels during Bay K 8644 intervention in depression-like models, we found that enhancing the expression of Dnmt3a could improve LPS-induced depression/anxiety-like behaviors, while inhibiting DNA methylation exacerbated anxiety-like behaviors, the combined use of BAY K 8644 and L-methionine can better improve depressive-like behavior. These findings indicate that DNA methylation plays a role in the regulation of depression-like behaviors by the L-type Ca^2+^ channel, and further research is needed to elucidate the interactions between DNA methylation and L-type Ca^2+^ channels.

## Introduction

Depression is currently one of the most common mental disorders, characterized by persistent low mood. Severe depression can even be life-threatening, significantly increasing the risk of suicide and having a profound negative impact on both physical and mental health (Whiteford et al., 2013). Presently, treatment for depression mainly focuses on pharmacotherapy and psychotherapy. Although pharmacotherapy has benefited many patients, a substantial proportion of individuals either do not respond to these medications or experience a delayed onset of therapeutic effects, resulting in inadequate treatment for many (Duman et al., 2016). Therefore, it is crucial to conduct in-depth research on the pathogenesis of depression to improve antidepressant therapies. Epidemiological studies indicate that approximately 40–50% of the risk for depression is hereditary (Fava and Kendler, 2000) and there is a mechanism between genetic factors and environmental factors that jointly affect the onset of depression.

The L-type Ca^2+^ channels mediates Ca^2+^ influx and associated regulatory events, playing a crucial role in the brain functions by regulating neuronal firing, activating Ca^2+^ signaling pathways involved in excitation-transcription coupling (ETC), and thus regulating neuronal plasticity associated with learning, memory, drug addiction, and neuronal development. Cav1,2 is the primary L-type Ca^2+^ channel expressed in the mammalian brain. In the mouse brain, quantitative polymerase chain reaction (qPCR) of RNA transcripts shows that Cav1,2 accounts for approximately 85% of LTCCs, while Cav1,3 constitutes most of the remaining portion (Sinnegger-Brauns et al., 2009).

Cacna1C is the gene encoding Cav1,2. Genome-Wide Association Studies (GWAS) have identified Cacna1C as a high-risk gene for psychiatric disorders. Cacna1C primarily encodes the Cav1,2 subunit of voltage-gated calcium channels, which is particularly associated with psychiatric conditions such as bipolar disorder (Ferreira et al., 2008; Moskvina et al., 2009; Clark et al., 2020). In rodent studies, knockout of Cacna1C in hippocampal and cortical neurons, 5-HT neuron-specific Cacna1C knockout, and Cacna1C heterozygous mice (Cacna1C ±) all exhibit anxiety-like behaviors (Ehlinger and Commons, 2019; Smedler et al., 2022). Cav1,2 activator BAY K 8644 can induce behavioral changes in rodents (Elliott et al., 2016). Therefore, the role of BAY K 8644 and Cav1,2 in depression requires further investigation.

DNA methylation, as a crucial epigenetic modification, plays a significant regulatory role in the pathogenesis of psychiatric disorders such as posttraumatic stress disorder (PTSD), depression, and autism (Liu et al., 2018). DNMTs (DNA methyltransferases) serve as vital regulators in the DNA methylation process, with Dnmt3a exhibiting direct catalytic activity and playing a critical role in mature neurons. Animal studies have revealed varying findings: mice experiencing depression due to social defeat exhibit lower levels of Dnmt3a expression in the medial prefrontal cortex compared to healthy controls, and up regulating Dnmt3a expression in this brain region can alleviate anxiety-like behaviors (Elliott et al., 2016). Conversely, research in rats demonstrated an increase in Dnmt3a and Dnmt3b expression in the medial prefrontal cortex of rats experiencing learned helplessness, with their expression levels down regulated by the antidepressant imipramine treatment (Sales and Joca, 2018). Additionally, variations in Dnmt3a expression in the ventral hippocampus of mice can play distinct roles in the development of depression in both males and females (Hodes et al., 2015). In our lab’s preliminary research, we observed that in a maternal separation-induced depression mouse model, Dnmt3a expression decreased in the hippocampus while increasing in the medial prefrontal cortex compared to control mice (Wang et al., 2022). These findings underscore the involvement of Dnmt3a in the development of depression, though the underlying mechanisms remain unclear.

The expression of Dnmt3a is correlated with changes in Ca^2+^ levels. DNA methyltransferase inhibitors can influence potassium channel activity by altering the expression of genes encoding proteins, participating in intracellular Ca^2+^ accumulation through voltage-gated Ca^2+^ channels entering or releasing from intracellular Ca^2+^ stores (Adelman et al., 2012). Studies in cardiovascular research have found that DNA methylation is involved in regulating tissue-specific expression of Cacna1C, and DNA methyltransferases may regulate the expression of Cacna1C (Zhao et al., 2022). Therefore, we hypothesize that DNA methylation and Cacna1C may jointly play a role in the process of depression.

In this study, we initially investigated the L-type Ca^2+^ channel activator BAY K 8644 in comparison to the antidepressant Venlafaxine using an LPS-induced depression model. Our results revealed that, compared to BAY K 8644, Venlafaxine exhibited superior improvement in depression/anxiety-like behaviors and increased Dnmt3a expression. Furthermore, we manipulated DNA methylation levels during BAY K 8644 intervention in the depression-like model, observing that simultaneous augmentation of DNA methylation alongside BAY K 8644 intervention ameliorated LPS-induced depression/anxiety-like behaviors, while inhibiting DNA methylation facilitated anxiety-like behaviors. These findings underscore the significant role of DNA methylation in regulating depression-like behaviors mediated by L-type Ca^2+^ channels. Additionally, the incorporation of methyl-donor supplements such as B vitamins alongside calcium channel modulation may offer promising benefits for ameliorating depression-like behaviors. However, further investigation is warranted to elucidate the interplay between DNA methylation and Cacna1C.

## Materials and methods

### Animal

C57BL/6 J mice were procured from SPF (Beijing) Biotechnology Co., Ltd. The mice were housed in accordance with standard laboratory conditions at a temperature of 22 ± 1°C and a 12-h light/dark cycle (lights on at 8 AM) at the Dali University Laboratory Animal Center. Recent studies have indicated that Dnmt3a expression is influenced by both sex (Nugent et al., 2015) and age (Feng et al., 2005; Wang et al., 2022). To control for these variables, we exclusively utilized male mice in all our experiments All mice aged 8 to 12 weeks. Each experimental group comprised mice from the same batch and age group. All behavioral experiments were conducted between 9 AM and 6 PM. The animal experiments adhered to the ARRIVE guidelines for the care and use of experimental animals and received approval from the Animal Care Ethical Committee of Dali University.

### Drugs

Mice in the LPS group received intraperitoneal injections of LPS (1 mg/kg, L2880, Sigma-Aldrich, USA) (Ali et al., 2020) for 7 consecutive days, with LPS administered 1 h prior to each behavioral test. Mice in the LPS + L-methionine group were fed with L-methionine chow (19.5 mg/kg, M5380, Sigma-Aldrich, USA) for 7 consecutive days (Miousse et al., 2017). Following 7 days of intraperitoneal LPS injections, mice in the LPS + BAY K 8644 group received BAY K 8644 (1 mg/kg, A8632, APE × BIO, USA) 1 h before each behavioral test (Shelton et al., 1987). Mice in the LPS + RG108 group and LPS + venlafaxine group were administered RG108 (0.2 mg/kg, R8279, Sigma-Aldrich, USA) and venlafaxine (2.5 mg/kg, A5355, APE × BIO, USA) respectively (Guo et al., 2020; Sales et al., 2021; Zheng et al., 2022), 2 h prior to each behavioral assessment, following 7 consecutive days of intraperitoneal LPS injections. Mice in the LPS + BAY K 8644 + LM group received mouse methionine chow alongside intraperitoneal LPS injections for 7 consecutive days, with mouse BAY K 8644 administered 1 h before each behavioral test. Mice in the RG108 group received intraperitoneal injections of RG108 2 h prior to each behavioral test. The Vehicle group received intraperitoneal injections of saline for seven consecutive days and were administered mouse saline 1 h before each behavioral test. LPS, venlafaxine, and BAY K 8644 were dissolved in saline, while RG108 was dissolved in DMSO and diluted in saline.

### Sucrose preference test

Indicators commonly used to evaluate pleasure deficit in depression modeling. The specific experimental protocol was as follows: the adaptation period of sucrose preference experiment was started on the fourth day of modeling, two roller ball water bottles were prepared, one bottle of 1% sucrose solution and one bottle of normal drinking water were prepared and placed in the rearing cages of the mice (during which the mice were fed with chow), the mice were free to choose, and the mice were placed in the cages continuously for 72 h (on the sixth day of the modeling), then the two bottles and the chow were withdrawn and the mice were fasted for 24 h. Behavioral tests were conducted on the seventh day of the modeling, and the mice were kept in a single cage for 24 h (during which they were fed with normal chow), and the positions of the water bottles were exchanged at 4hr intervals to prevent the formation of positional preference. The consumption of drinking water and sucrose solution was recorded respectively after 24 h. The test index was: sugar water preference index = sucrose solution consumption/total consumption (tap water + sucrose solution) × 100%.

### Forced swimming test

The forced swimming test is commonly used to evaluate indicators of behavioral despair in depression models. The specific experimental protocol was as follows: after the sucrose preference test, the mice tested individually were returned to the home cage and placed into the behavioral chamber for acclimatization at least 1hr prior to the start of the forced swimming test, and the room temperature of the behavioral chamber was maintained at 25°C. Prepare a cylindrical transparent acrylic water tank with a diameter of 15cm and a height of 30cm in advance, and fill the water tank with pure water; and the water temperature was controlled at 25 ± 2°C. Place the mice in the tank and start the test after 2 min of adaptation, the test duration is 3 min, and the floating time (i.e., the time when the mouse stops struggling and its body floats on the water surface) is filmed and recorded with the animal behavior analysis equipment. Note that each mouse needed to be replaced with clean tap water to prevent feces and urine from interfering with the behavior of the mice to be tested. (Control and modeling groups were alternated throughout the testing period).

### Open field test

The open field experiment is mainly used to assess the locomotion level and anxiety level of mice. The size of the open field used was 45 × 45 × 30 cm with a brightness of about 150 lux. The mice were taken out from the feeding room and acclimatized in the behavioral room for at least 1hr before starting the experiment. During the experiment, the total activity of the mice was recorded for 15 min, and the data was collected using Visual Track software. At the end of the experiment, the same cage was used to transfer the mice to another empty cage in the acclimatization room, and all the mice in the same cage were put back to the home cage together when they were done. The Vehicle group received intraperitoneal injections of saline for seven consecutive days and were administered mouse saline 1 h before each behavioral test (Yue et al., 2023). The absent field was scrubbed with 75% ethanol in the middle of every 2 mice. The room temperature of the behavioral room was maintained at 25°C.

### Immunofluorescence staining

Mice were anesthetized with sodium pentobarbital, perfused transcardially with 4% paraformaldehyde and PBS, and sectioned at 30 μm. Brain sections were treated with 0.5% Triton X-100, 10% goat serum, and 0.2% skimmed milk powder in PBS for 1 h at room temperature. Sections were incubated overnight at room temperature with primary antibodies (anti-Dnmt3a (rabbit), 1:1000, CST, #3598; anti-GFAP (rabbit), 1:500, MCE, #264369). The slices were incubated overnight at room temperature, washed three times with PBS (10 min each time), incubated in the dark with secondary antibodies (goat anti-rabbit 488, A11008, 1:400, Invitrogen) for 1 h at room temperature, and washed three times with PBS (10 min each time). Finally, sections were incubated with Hoechst for 5 min and mounted on glass slides. Images were captured using a 10 × (ix73; Olympus, Japan) inverted microscope. The co-localization of Dnmt3a-positive cells was counted using Image J.

### Data analysis

At least 3 brain slices were taken from each DG region, as shown in The Mouse brain in Stereotaxic Coordinates, and counted according to Image J. all the images analysis was made using ImageJ and statistical analysis using GraphPad Prism8. Stat significance was set at *P* < 0.05, all the data are expressed as mean ± SEM.

## Results

### LPS induces depressive-like behavior and reduces Dnmt3a expression in the dentate gyrus (DG) region of the mouse hippocampus

**FIGURE 1:**
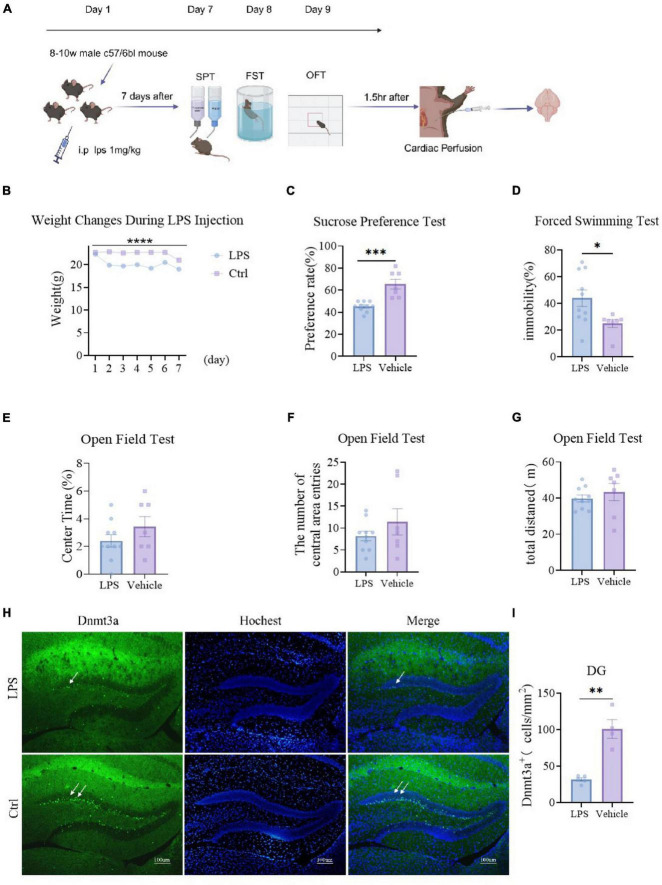
LPS induces depressive-like behavior and reduces Dnmt3a expression in the dentate gyrus (DG) region of the mouse hippocampus. **(A)** Experimental procedures. Ctrl group, *n* = 7; LPS group, *n* = 10. **(B)** Weight changes of the two groups of mice, The weight of the LPS group was significantly lower than that of the control group (two-way RM ANOVA, F _(1_, _15)_ = 47.26, *P* < 0.0001). **(C)** Sucrose preference test results showed that the sucrose preference of the LPS group was significantly lower than that of the control group (unpaired t-test, T_15_ = 5.031, *P* = 0.0001). **(D)** Forced swim test results, the immobility time of the LPS group was significantly higher than that of the control group (unpaired *t*-test, T_15_ = 2.391, P = 0.030) **(E–G)** Open field test results. **(E)** Percentage of time in the center area (LPS group vs. Ctrl group; unpaired t-test, T_15_ = 1.276, *P* = 0.221); **(F)** the number of central area entries (LPS group vs. Ctrl group; unpaired t-test, T_15_ = 1.157, *P* = 0.266); **(G)** total distance (LPS group vs. Ctrl group; unpaired t-test, T_15_ = 0.758, *P* = 0.459). **(H)** Sample Images of Dnmt3a staining in the hippocampal dentate gyrus. Scale bars, 100 μm. **(I)** Quantification of Dnmt3a expression in the hippocampal dentate gyrus. (LPS group, *n* = 5; Ctrl group, *n* = 4; unpaired t-test, T_7_ = 6.004, *P* = 0.0005). Results are presented as mean ± SEM; *P < 0.05; **P < 0.01; ***P < 0.001, ****P < 0.0001; ns, not significant.

We administered LPS intraperitoneally to mice for 7 consecutive days, monitored their body weight (Figure 1B, *P* < 0.0001), conducted behavioral tests, and subsequently perfused and stained their brains (Figure 1A). Results showed a significant decrease in body weight in the LPS group compared to the Ctrl group (*P* < 0.0001) (Figure 1B). The sucrose preference test revealed a significant reduction in sucrose consumption in the LPS group compared to the Ctrl group (*P* = 0.0001) (Figure 1C). In the forced swimming test, the floating time of the modeled group was significantly lower than that of the control (*P* = 0.030) (Figure 1D). In the open field test, the modeled group showed no significantly change compared to the control group on total distance (*P* = 0.459) (Figure 1G). compared to the LPS group, the Ctrl group exhibited an increasing trend but not significantly in both the percentage of time spent in the central area (*P* = 0.221) and the number of entries (*P* = 0.066) (Figures 1E, F). These behavioral findings collectively demonstrate that LPS significantly increases depressive-like behavior in mice.

Under physiological and pathological conditions, the regulation of neurogenesis in the hippocampal DG region significantly affects mechanisms involved in emotion, memory, and cognition. Previous studies have shown that Dnmt3a is primarily expressed in the adult mouse DG region (Gong and Zhou, 2018; Wang et al., 2022). Therefore, we primarily stained the DG region for Dnmt3a. We randomly selected 5 mice from the same group for immunofluorescence staining and analysis of Dnmt3a. The results showed that the expression of Dnmt3a in the LPS group was significantly decreased compared with the control group (*P* = 0.0005) (Figures 1H, I). This result indicated that while LPS induced depression in mice, the level of Dnmt3a in the DG region also decreased. Abnormal levels of Dnmt3a affect depressive behavior in mice, which is consistent with our previous findings (Wang et al., 2022).

### Compared to BAY K 8644, venlafaxine shows better improvement in depressive/anxiety-like behaviors and can reverse the decrease in DNMT3a expression induced by LPS injection

**FIGURE 2:**
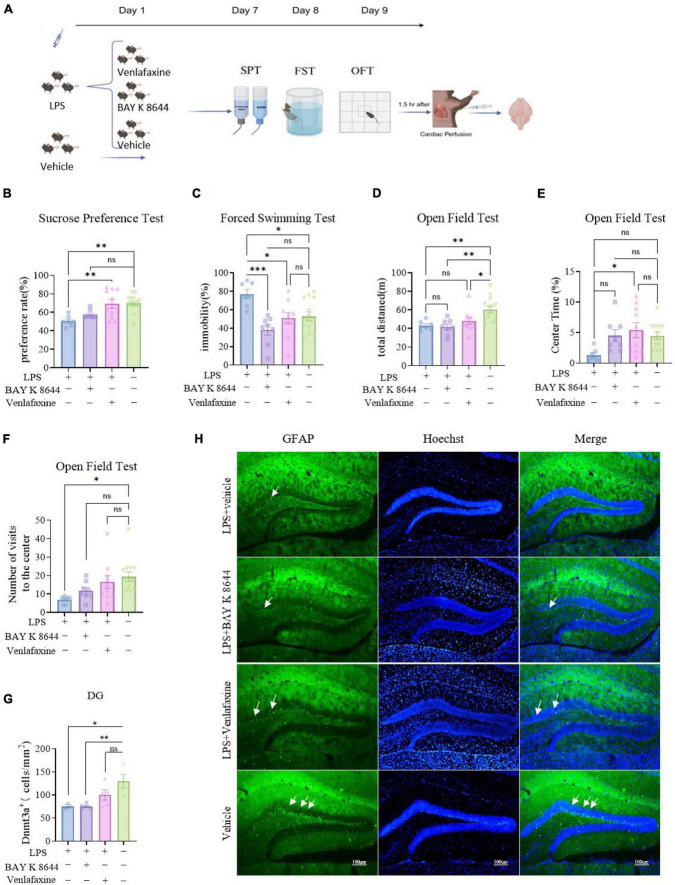
Compared to BAY K 8644, venlafaxine shows better improvement in depressive/anxiety-like behaviors and can reverse the decrease in DNMT3a expression induced by LPS injection. **(A)** Experimental procedures. Ctrl group, *n* = 13; LPS group, *n* = 7; LPS + BAY K 8644 group, *n* = 8; LPS + Venlafaxine group, *n* = 10. **(B)** Sucrose preference test results (one-way ANOVA with Tukey’s multiple comparisons test, *F*_(3_, _34)_ = 7.172, *P* = 0.0007; LPS vs. LPS + BAY K 8644, T_34_ = 1.881, *P* = 0.551; LPS vs. LPS + Venlafaxine, T_34_ = 5.230, *P* = 0.004; LPS vs. Ctrl, T_34_ = 5.601, *P* = 0.002; LPS + BAY K 8644 vs. LPS + Venlafaxine, T_34_ = 3.381, *P* = 0.098; LPS + BAY K 8644 vs. Ctrl, T_34_ = 3.676, *P* = 0.063; LPS + Venlafaxine vs. Ctrl, T_34_ = 0.115, *P* = 0.999). **(C)** Forced swim test results (one-way ANOVA with Tukey’s multiple comparisons test, F_(3_, _34)_ = 6.722, *P* = 0.001; LPS vs. LPS + BAY K 8644, T_34_ = 6.249, *P* = 0.0005; LPS vs. LPS + Venlafaxine, T_34_ = 4.400, *P* = 0.019; LPS vs. Ctrl, T_34_ = 4.295, *P* = 0.023; LPS + BAY K 8644 vs. LPS + Venlafaxine, T_34_ = 2.247, *P* = 0.398; LPS + BAY K 8644 vs. Ctrl, T_34_ = 2.715, *P* = 0.239; LPS + Venlafaxine vs. Ctrl, T_34_ = 0.367, *P* = 0.994). **(D–F)** Open field test results. **(D)** total distance (one-way ANOVA with Tukey’s multiple comparisons test, F_(3_, _34)_ = 6.735, *P* = 0.001; LPS vs. LPS + BAY K 8644, T_34_ = 0.3923, *P* = 0.992; LPS vs. LPS + Venlafaxine, T_34_ = 1.341, *P* = 0.779; LPS vs. Ctrl, T_34_ = 4.844, *P* = 0.008; LPS + BAY K 8644 vs. LPS + Venlafaxine, T_34_ = 1.821, *P* = 0.577; LPS + BAY K 8644 vs. Ctrl, T_34_ = 5.506, *P* = 0.002; LPS + Venlafaxine vs. Ctrl, T_34_ = 3.828, *P* = 0.049) **(E)** Percentage of time in the center area (one-way ANOVA with Tukey’s multiple comparisons test, F_(3_, _34)_ = 3.505, P = 0.026; LPS vs. LPS + Venlafaxine, T_34_ = 4.412, *P* = 0.018); **(F)** the number of central area entries (one-way ANOVA with Tukey’s multiple comparisons test, *F*_(3_, _34)_ = 4.034, *P* = 0.015; LPS vs. Ctrl, T_34_ = 4.597, *P* = 0.013); **(H)** Sample Images of Dnmt3a staining in the hippocampal dentate gyrus. Scale bars, 100μm. **(G)** Quantification of Dnmt3a expression in the hippocampal dentate gyrus. (Ctrl group, *n* = 4; LPS group, *n* = 3; LPS + BAY K 8644 group, *n* = 5; LPS + Venlafaxine group, n = 5; one-way ANOVA with Tukey’s multiple comparisons test, F_(3_, _13)_ = 6.666, *P* = 0.006; LPS vs. LPS + BAY K 8644, T_13_ = 0.0006, P > 0.999; LPS vs. LPS + Venlafaxine, T_13_ = 2.388, P = 0.368; LPS vs. Ctrl, T_13_ = 5.021, P = 0.016; LPS + BAY K 8644 vs. LPS + Venlafaxine, T_13_ = 2.758, *P* = 0.256; LPS + BAY K 8644 vs. Ctrl, T_34_ = 5.718, *P* = 0.007; LPS + Venlafaxine vs. Ctrl, T_13_ = 3.117, *P* = 0.173). Results are presented as mean ± SEM; *P < 0.05; **P < 0.01; ***P < 0.001; ns, not significant.

Mice subjected to consecutive LPS injections were divided into three groups. One hour before behavioral testing, they were administered with venlafaxine/Cacna1C activator BAY K 8644/vehicle, followed by behavioral testing. After behavioral testing, brains were collected for Dnmt3a staining (Figure 2A). The sucrose preference test results showed that venlafaxine injection could counteract the decreased sucrose preference induced by LPS, whereas BAY K 8644 could not, LPS + venlafaxine showed no difference in sucrose preference compared to the control group (*P* = 0.999), while LPS + BAY K 8644 showed tend lower preference (*P* = 0.063) and LPS showed significantly differences (*P* = 0.002) (Figure 2B). Forced swimming test results indicated that both venlafaxine and BAY K 8644 could eliminate the despair behavior induced by LPS in the forced swimming test, the immobility time of the LPS + venlafaxine (*P* = 0.0994) and LPS + BAY K 8644 groups (*P* = 0.239) showed no significant difference from the control group, while the control and LPS groups had significant differences (*P* = 0.023) (Figure 2C). Open field test results demonstrated that the total distance traveled by mice in the LPS group (*P* = 0.008), LPS + venlafaxine group (*P* = 0.049) and LPS + BAY K 8644 group (*P* = 0.002) decreased compared to the control group (Figure 2D). The number of central zone visits and time spent in the central zone showed no difference between the LPS + venlafaxine group, LPS + BAY K 8644 group, while they were significantly reduced in the LPS group compared to the control group (Figures 2E, F). Behavioral results indicate that BAY K 8644 can alleviate depressive-like behavior induced by LPS to some extent, but its antidepressant effect is not as significant as venlafaxine.

**FIGURE 3:**
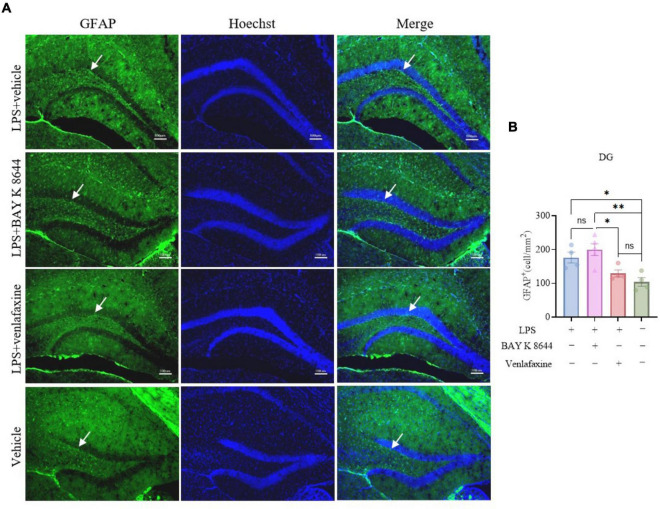
Venlafaxine reverse the increase expression of GFAP in the hippocampal dentate gyrus. **(A)** Sample images of GFAP staining in the hippocampal dentate gyrus. Scale bars, 100μm. **(B)** Quantification of GFAP expression in the hippocampal dentate gyrus. (Ctrl group, *n* = 4; LPS group, *n* = 3; LPS + BAY K 8644 group, *n* = 5; LPS + Venlafaxine group, *n* = 5; one-way ANOVA with Tukey’s multiple comparisons test, *F*_(3_, _13)_ = 8.528, *P* = 0.002; LPS vs. LPS + BAY K 8644, T_13_ = 1.568, *P* = 0.691; LPS vs. LPS + Venlafaxine, T_13_ = 3.033, *P* = 0.190; LPS vs. Ctrl, T_13_ = 4.646, *P* = 0.027; LPS + BAY K 8644 vs. LPS + Venlafaxine, T_13_ = 4.765, *P* = 0.023; LPS + BAY K 8644 vs. Ctrl, T_34_ = 6.465, *P* = 0.003; LPS + Venlafaxine vs. Ctrl, T_13_ = 1.613, *P* = 0.672). Results are presented as mean ± SEM; *P < 0.05; **P < 0.01; ns, not significant.

Dnmt3a staining results showed that compared to the control group, both the LPS group (*P* = 0.016) and LPS + BAY K 8644 group (*P* = 0.007) exhibited a significant decrease in Dnmt3a expression (Figures 2G, H), while the LPS + venlafaxine group (*P* = 0.173) showed no difference. GFAP is a marker of astrocytes whose expression is associated with inflammation (Kunchok et al., 2019). We also performed GFAP staining, and the results showed that compared to the control group, the expression of GFAP increased significantly in the LPS + BAY K 8644 group (*P* = 0.003), while there was no difference in the LPS + venlafaxine group (*P* = 0.672) (Figures 3A, B). These results suggest that compared to BAY K 8644, venlafaxine shows better improvement in depressive/anxiety-like behaviors and can reverse the decrease in Dnmt3a expression and the increase in inflammation induced by LPS injection.

### The regulation in Dnmt3a levels can impact depressive-like behavior in mice, and the combined use of BAY K 8644 and L-methionine can better improved depressive-like behaviors

**FIGURE 4:**
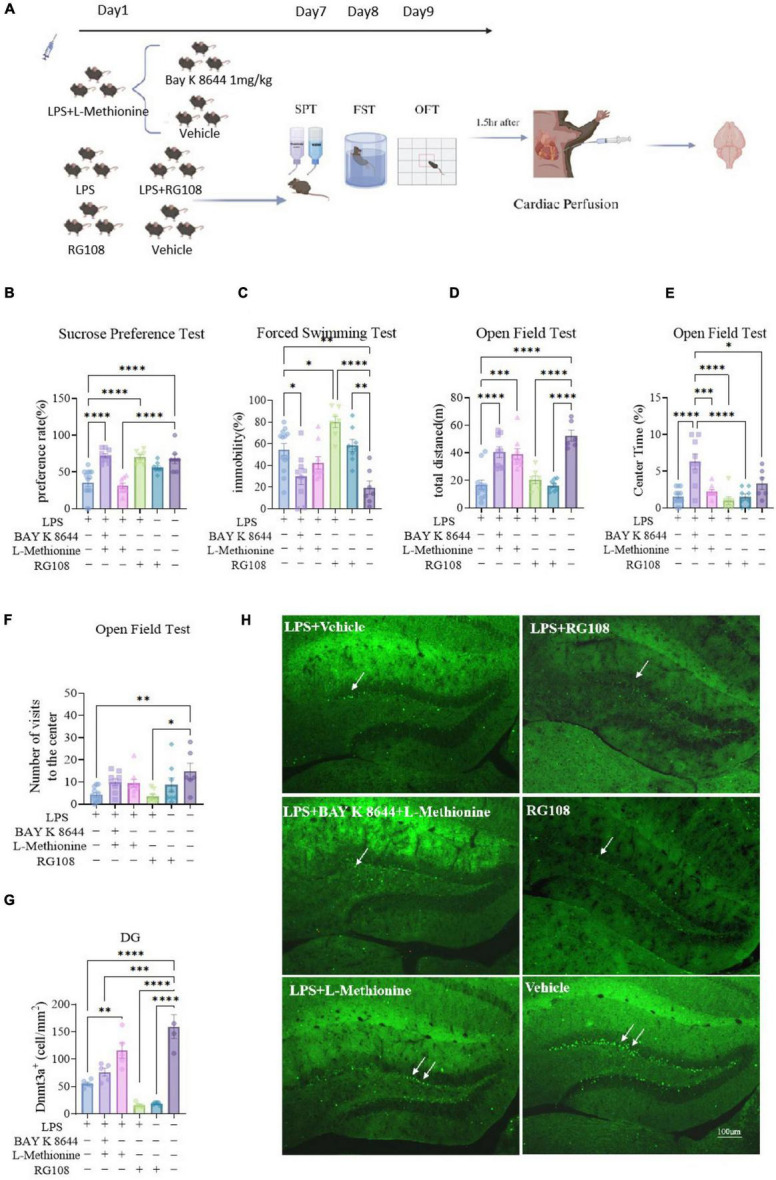
The regulation in Dnmt3a levels can impact depressive-like behavior in mice, and the combined use of BAY K 8644 and L-methionine can better improved depressive-like behaviors. **(A)** Experimental procedures. Ctrl group, *n* = 7; LPS group, *n* = 13; LPS + BAY K 8644 + LM group, *n* = 9; LPS + LM group, *n* = 9; LPS + RG108 group, *n* = 7; RG108 group, *n* = 8. **(B)** Sucrose preference test results (one-way ANOVA with Tukey’s multiple comparisons test, *F*_(5_, _47)_ = 16.71, *P* < 0.0001; LPS vs. LPS + BAY K 8644 + LM, T_47_ = 8.789, *P* < 0.0001; LPS vs. LPS + RG108, T_47_ = 7.778, *P* < 0.0001; LPS vs. RG108, T_47_ = 4.739, *P* = 0.019; LPS vs. Ctrl, T_47_ = 7.226, *P* < 0.0001; LPS + BAY K 8644 + LM vs. LPS + LM, T_47_ = 9.072, *P* < 0.0001; LPS + LM vs. LPS + RG108, T_47_ = 8.159, *P* = 0.006; LPS + LM vs. Ctrl, T_47_ = 7.646, *P* < 0.0001). **(C)** Forced swim test results(one-way ANOVA with Tukey’s multiple comparisons test, *F*_(5_, _47)_ = 10.66, *P* < 0.0001; LPS vs. LPS + BAY K 8644 + LM, T_47_ = 4.465, *P* = 0.031; LPS vs. LPS + RG108, T_47_ = 4.214, *P* = 0.049; LPS vs. Ctrl, T_47_ = 5.848, *P* = 0.002; LPS + BAY K 8644 + LM vs. LPS + RG108, T_47_ = 7.762, *P* < 0.0001; LPS + BAY K 8644 + LM vs. RG108, T_47_ = 4.576, *P* = 0.025; LPS + LM vs. LPS + RG108, T_47_ = 5.817, *P* = 0.002; LPS + RG108 vs. Ctrl, T_47_ = 8.825, P < 0.0001; RG108 vs. Ctrl, T_47_ = 5.853, *P* = 0.002). **(D–F)** Open field test results. Ctrl group, n = 6; LPS group, n = 12; LPS + BAY K 8644 + LM group, *n* = 9; LPS + LM group, *n* = 9; LPS + RG108 group, *n* = 7; RG108 group, *n* = 8. **(D)** Total distance (one-way ANOVA with Tukey’s multiple comparisons test, *F*_(5_, _45)_ = 17.98, *P* < 0.0001). **(E)** Percentage of time in the center area (one-way ANOVA with Tukey’s multiple comparisons test, *F*_(5_, _45)_ = 11.26, *P* < 0.0001); **(F)** the number of central area entries (one-way ANOVA with Tukey’s multiple comparisons test, *F*_(5_, _45)_ = 3.936, *P* = 0.005); **(H)** Sample Images of Dnmt3a staining in the hippocampal dentate gyrus. Scale bars, 100μm. **(G)** Quantification of Dnmt3a expression in the hippocampal dentate gyrus. (Ctrl group, n = 4; LPS group, *n* = 5; LPS + BAY K 8644 + LM group, n = 5; LPS + LM group, n = 5; LPS + RG108 group, *n* = 5; RG108 group, *n* = 5; one-way ANOVA with Tukey’s multiple comparisons test, *F*_(5_, _23)_ = 30.11, *P* < 0.0001). Results are presented as mean ± SEM; *P < 0.05; **P < 0.01; ***P < 0.001; ****P < 0.0001; ns, not significant.

To modulate the expression levels of Dnmt3a in the mouse brain, we utilized the Dnmt3a inhibitor RG108 and the DNA methylation substrate L-methionine. In a pilot experiment, we found that feeding mice with L-methionine added to their diet continuously for 7 days significantly increased the level of Dnmt3a expression. In this experiment, we initiated a high-methylation diet concurrently with the start of LPS administration, and then administered Bay K 8644 (LPS + LM + BAY K 8644 group) or vehicle (LPS + LM group) 1 h before behavioral testing. To investigate the impact of reduced methylation on LPS-induced depressive-like behavior, we also included two groups of mice: one receiving the DNA methyltransferase inhibitor RG108 before behavioral testing following LPS injection (LPS + RG108 group) and another receiving RG108 but not LPS (RG108 group). These mice were compared with a control group injected only with vehicle (ctrl group) (Figure 4A).

Behavioral results showed that in the sucrose preference test, compared to the ctrl group, mice in the LPS (*P* < 0.0001) and LPS + LM (*p* < 0.0001) groups exhibited significantly reduced sucrose preference, while the remaining groups showed no significant differences compared to the control group (Figure 4B). In the forced swim test, compared to the ctrl group, mice in the LPS (*P* = 0.002), LPS + RG108 (*P* < 0.0001), and RG108 (*P* = 0.002) groups all exhibited significantly increased immobility time in the forced swim, while the other groups showed no differences compared to the control group (Figure 4C). In the open field test, compared to the control group, the LPS (*P* < 0.0001), LPS + RG108 (*P* < 0.0001), and RG108 (*P* < 0.0001) groups all displayed reduced locomotor activity, while the LPS + LM + BAY K 8644 (*P* = 0.223) and LPS + LM (*P* = 0.139) groups showed no significant differences compared to the control group (Figure 4D); the LPS (*P* = 0.008) and LPS + RG108(*P* = 0.012) groups exhibited reduced entries into the central zone (Figure 4F), while the LPS + LM + BAY K 8644 group (*P* = 0.023) showed significantly increased time spent in the central zone compared to the control group (Figure 4E). These results indicate that L-methionine and BAY K 8644 can significantly improve depressive-like behavior in LPS-treated mice, with L-methionine alone partially improving depressive-like behavior (reduced immobility time in the forced swim test and increased locomotor activity and entries into the central zone in the open field test compared to the LPS group). Additionally, RG108 injection exhibited increased depressive-like behavior in the forced swim test, and even without LPS injection, it led to increased depressive-like behavior in the open field and forced swim tests. However, in the sucrose preference test, the RG108 group showed normal sucrose preference. This suggests that DNA methyltransferase inhibitors can increase some aspects of depressive-like behavior, while increasing methylation substrates can ameliorate depressive-like behavior. The best antidepressant effect was achieved when increasing methylation substrates while administering BAY K 8644.

Dnmt3a staining results showed that the Dnmt3a expression levels in the LPS + LM + BAY K 8644 (*P* = 0.0001), RG108 + LPS (*P* < 0.0001), and RG108 (*P* < 0.0001) groups were significantly lower than those in the control group (Figures 4G, H). These staining results indicate that a high-methylation diet and RG108 injection indeed play a role in modulating Dnmt3a expression levels in the hippocampal region. And the combined use of BAY K 8644 and L-methionine can’t increase the expression of Dnmt3a.

These results demonstrate that changes in Dnmt3a levels can influence depressive-like behavior in mice, and the combined use of BAY K 8644 and L-methionine can better improve depressive-like behavior. However, the combined use of BAY K 8644 and L-methionine does not significantly improve the decrease in Dnmt3a expression induced by LPS, suggesting that there may be a more complex mutual regulation between Dnmt3a and Cacna1C in the regulation of depression.

## Discussion

In this study, we investigated the L-type Ca^2+^ channel activator BAY K 8644 in comparison with the antidepressant Venlafaxine in a depressive model induced by LPS. Our results demonstrate that Venlafaxine is more effective than BAY K 8644 in ameliorating depression like behaviors and increasing Dnmt3a expression while decreasing GFAP expression. Furthermore, we manipulated DNA methylation levels during BAY K 8644 intervention in the depressive model, and found that increasing DNA methylation simultaneously with BAY K 8644 intervention could ameliorate LPS-induced depressive/anxiety-like behaviors, while inhibiting DNA methylation could promote anxiety-like behaviors. Our findings suggest a potential interaction between Dnmt3a and L-type Ca^2+^ channels in the context of depression.

Continuous LPS injection in mice has been shown to induce depressive-like behaviors including increased immobility in forced swimming and tail suspension tests, and decreased sucrose preference (Frenois et al., 2007). Additionally, the LPS-induced depressive model is associated with changes in brain regions such as the hippocampus, amygdala, and hypothalamus, primarily modeling the inflammation hypothesis of depression (Frenois et al., 2007). Consistent with our previous research showing predominant Dnmt3a expression in the dentate gyrus (DG) region of adult mice (Wang et al., 2022), we found a decrease in Dnmt3a expression in the DG region of mice exhibiting LPS-induced depressive-like behaviors. In 2018, Stephanie M Matt et al. discovered that the DNA methyltransferase inhibitor zebularine could affect the expression changes of inflammation-related genes induced by LPS, altering the course of LPS-induced inflammation (Matt et al., 2018). Studies in the cardiovascular system have found that inhibiting Dnmt3a expression in bone marrow cells can lead to an increase in inflammation and the severity of atherosclerosis in mice (Rauch et al., 2023). These results collectively suggest an association between Dnmt3a and chronic inflammation, with decreased Dnmt3a expression potentially affecting the progression of inflammatory responses. Therefore, increasing Dnmt3a expression may improve LPS-induced inflammatory responses, consistent with our GFAP staining results.

Venlafaxine not only improves depressive-like behaviors but also reverses the downregulation of Dnmt3a expression and the upregulation of GFAP expression induced by LPS. This suggests that Venlafaxine may elevate DNA methylation levels while improving inflammation. Clinical studies have shown that the use of venlafaxine can reduce GFAP levels in the cerebrospinal fluid of patients with depression (Zheng et al., 2023), consistent with our staining results. There have been no reports on the relationship between venlafaxine and Dnmt3a, we speculate that venlafaxine may influence Dnmt3a expression through its effects on inflammation.

BAY K 8644 is an L-type Ca^2+^ channel activator. As early as 1985, P. Skolnick et al. found that injection of 2–4 mg/kg BAY K 8644 could induce behavioral changes in mice, including ataxia, decreased motor activity, Straub tail, arched back, limb clonus and tonus, and an increased sensitivity to auditory stimulation (Bolger et al., 1985). Similar behavioral changes were observed in rats with a dose of 2 mg/kg BAY K 8644 (Baran et al., 2000). In 2002, Kasim et al. reported that doses ranging from 2 to 8 mg/kg of BAY K 8644 could induce self-biting behavior in mice by affecting serotonin (Kasim et al., 2002). Additionally, research has shown that doses of 0.5 mg/kg and 1 mg/kg BAY K 8644 can prolong immobility time in the despair test in mice (Mogilnicka et al., 1988). In rat studies, it was found that using 0.5 mg/kg BAY K 8644 alone did not affect anxiety-like behavior, but when combined with ethanol, it enhanced the anxiolytic effects of ethanol (Kirac and Eroglu, 1991). In our study, we found that a dose of 1 mg/kg BAY K 8644 reduced despair behavior in the forced swim test in the LPS model mice, increased central access time and frequency in the open field test, but did not significantly affect sucrose preference. The role of the L-type Ca^2+^ channel-encoding gene Cacna1C in psychiatric disorders also exhibits variability. Most studies suggest that Cacna1C is a highly risk-associated gene for psychiatric disorders, with its overexpression leading to emotional, cognitive, and memory deficits. For example, heterozygous Cacna1C knockout mice exhibit social deficits similar to humans (Kabitzke et al., 2018), and selective deletion of Cacna1C in the prefrontal cortex also shows antidepressant effects (Kabir et al., 2017). However, Ehlinger and Commons also confirmed that selective deletion of Cacna1C can increase immobility time in the forced swim test and anxiety levels in the open field test (Ehlinger and Commons, 2019). These studies suggest differences in the regulation of behavior by BAY K 8644 and Cacna1C in different experiments and contexts.

Higher or lower levels of Ca^2+^ influx in neurons may be detrimental. L-type Ca^2+^ channels can regulate neuronal firing. For example, L-type Ca^2+^ channels can directly provide depolarizing stimuli, which can stabilize depolarization plateaus, thereby affecting neuronal firing (Olson et al., 2005). L-type Ca^2+^ channels can also couple with Ca^2+^-activated potassium channels to regulate neuronal firing. Therefore, the regulation of behavior by BAY K 8644 may be related to its effects on L-type Ca^2+^ channels, as the level of channel opening directly affects behavioral changes (Berkefeld et al., 2006). Thus, the behavioral regulatory effects of BAY K 8644 may depend greatly on the purity of the drug, dosage regimen, and timing of administration. It may be the key point limiting the application of BAY K 8644.

Mice with depressive behavior due to social failure showed lower expression levels of Dnmt3a in the medial prefrontal cortex than healthy control mice. Increasing the expression of Dnmt3a in the medial prefrontal cortex can reduce anxiety-like behavior (Elliott et al., 2016). These studies, along with our findings of increased Dnmt3a expression levels in the LPS model mice through dietary supplementation with L-methionine, reversing the increased immobility time in the forced swimming test and reduced central visits in the open field test induced by LPS, are consistent with previous research results. However, in the sucrose preference test, RG108 reversed the phenotype of reduced sucrose preference induced by LPS, which differed from the phenotypes observed in the forced swimming and open field tests. Administration of RG108 alone increased immobility time in the forced swimming test but did not affect the sucrose preference test. This difference may be related to the perception of sweet taste and signal transduction. Studies have shown that the methylation level of TAS1R2, a gene related to sweet taste transduction, is associated with sweet taste perception and preference (Ramos-Lopez et al., 2018). Therefore, RG108 may affect sweet taste perception by influencing the methylation level of TAS1R2, leading to differences in the sucrose preference test and other test results.

The reasons for the synergistic effect of L-methionine and BAY K 8644 in achieving better antidepressant effects may be twofold. On the one hand, DNA methylation may participate in the regulation of cacna1C gene expression. On the other hand, both are involved in the regulation of depression-related neural functions. L-type Ca^2+^ channels can regulate spine density in the CA1 region through the cAMP/PKA signaling pathway, affecting neural function (Hoogland and Saggau, 2004). Studies have found that increased expression of Dnmt3a in the nucleus accumbens (NAc) can increase spine density in the NAc, while RG108 can reduce spine density in cultured neurons (LaPlant et al., 2010). These studies suggest that both L-type Ca^2+^ channels and DNA methylation can affect spine density, and spine density is an important influencing factor in the regulation of depression. Regulation of the number, size, and shape of dendritic spines is important for synaptic plasticity, learning, and memory (Lai and Ip, 2013). In chronic stress depression models, spine density in the hippocampal area is reduced (Qiao et al., 2016), and reduced spine density in the hippocampal area is also found in LPS-induced depression models (Gong et al., 2023). These results suggest that DNA methylation and L-type Ca^2+^ channels may synergistically regulate spine density in the LPS model to improve depressive-like behavior.

Our study suggests that DNA methylation and L-type Ca^2+^ channels play regulatory roles in inflammation-induced depressive-like behavior. Activation of L-type Ca^2+^ channels alone and increasing Dnmt3a expression alone can alleviate depressive-like behavior to some extent, but their synergistic effect can enhance antidepressant efficacy. The specific mechanism of interaction is still unclear. Our results suggest that research on the synergistic effects of epigenetics and calcium ion channels may be helpful in developing new antidepressant therapies.

### Permission to reuse and copyright

The submitted manuscript (including the text, tables, figures, graphs, images, and any other related content) is original and has not been submitted to another journal for publication, has not been published before in whole or in part. The authors guarantee that the article does not infringe any personal or property right of others and accept the responsibility of the content of this manuscript and all other legal responsibilities related to the manuscript.

## Data Availability

The original contributions presented in the study are included in the article/supplementary material, further inquiries can be directed to the corresponding author.

## References

[B1] Adelman J. P. Maylie J. Sah P. (2012). Small-conductance Ca2+-activated K+ channels: form and function. *Annu. Rev. Physiol.* 74 245–269. 21942705 10.1146/annurev-physiol-020911-153336

[B2] Ali T. Rahman S. U. Hao Q. Li W. Liu Z. Ali Shah F. (2020). Melatonin prevents neuroinflammation and relieves depression by attenuating autophagy impairment through FOXO3a regulation. *J. Pineal Res.* 69 e12667. 10.1111/jpi.12667 32375205

[B3] Baran H. Kepplinger B. Hortnagl H. (2000). Clonidine modulates BAY K 8644-induced rat behavior and neurotransmitter changes in the brain. *Eur. J. Pharmacol.* 401 31–37. 10.1016/s0014-2999(00)00404-0 10915834

[B4] Berkefeld H. Sailer C. A. Bildl W. Rohde V. Thumfart J. O. Eble S. (2006). BKCa-Cav channel complexes mediate rapid and localized Ca2+-activated K+ signaling. *Science* 314 615–620. 10.1126/science.1132915 17068255

[B5] Bolger G. T. Weissman B. A. Skolnick P. (1985). The behavioral effects of the calcium agonist Bay K 8644 in the mouse: antagonism by the calcium antagonist nifedipine. *Naunyn. Schmiedebergs. Arch. Pharmacol.* 328 373–377. 2581145 10.1007/BF00692903

[B6] Clark M. B. Wrzesinski T. Garcia A. B. Hall N. A. L. Kleinman J. E. Hyde T. (2020). Long-read sequencing reveals the complex splicing profile of the psychiatric risk gene CACNA1C in human brain. *Mol. Psychiatry* 25 37–47. 10.1038/s41380-019-0583-1 31695164 PMC6906184

[B7] Duman R. S. Aghajanian G. K. Sanacora G. Krystal J. H. (2016). Synaptic plasticity and depression: new insights from stress and rapid-acting antidepressants. *Nat. Med.* 22 238–249. 26937618 10.1038/nm.4050 PMC5405628

[B8] Ehlinger D. G. Commons K. G. (2019). Cav1.2 L-type calcium channels regulate stress coping behavior via serotonin neurons. *Neuropharmacology* 144 282–290. 30176250 10.1016/j.neuropharm.2018.08.033 PMC7476513

[B9] Elliott E. Manashirov S. Zwang R. Gil S. Tsoory M. Shemesh Y. (2016). Dnmt3a in the Medial Prefrontal Cortex Regulates Anxiety-Like Behavior in Adult Mice. *J. Neurosci.* 36 730–740. 10.1523/JNEUROSCI.0971-15.2016 26791204 PMC6601996

[B10] Fava M. Kendler K. S. (2000). Major depressive disorder. *Neuron* 28 335–341. 11144343 10.1016/s0896-6273(00)00112-4

[B11] Feng J. Chang H. Li E. Fan G. (2005). Dynamic expression of de novo DNA methyltransferases Dnmt3a and Dnmt3b in the central nervous system. *J. Neurosci. Res.* 79 734–746. 10.1002/jnr.20404 15672446

[B12] Ferreira M. A. O’Donovan M. C. Meng Y. A. Jones I. R. Ruderfer D. M. Jones L. (2008). Collaborative genome-wide association analysis supports a role for ANK3 and CACNA1C in bipolar disorder. *Nat. Genet.* 40 1056–1058. 10.1038/ng.209 18711365 PMC2703780

[B13] Frenois F. Moreau M. O’Connor J. Lawson M. Micon C. Lestage J. (2007). Lipopolysaccharide induces delayed FosB/DeltaFosB immunostaining within the mouse extended amygdala, hippocampus and hypothalamus, that parallel the expression of depressive-like behavior. *Psychoneuroendocrinology* 32 516–531. 10.1016/j.psyneuen.2007.03.005 17482371 PMC1978247

[B14] Gong Q. Li W. Ali T. Hu Y. Mou S. Liu Z. (2023). eIF4E phosphorylation mediated LPS induced depressive-like behaviors via ameliorated neuroinflammation and dendritic loss. *Transl. Psychiatry* 13 352. 10.1038/s41398-023-02646-5 37978167 PMC10656522

[B15] Gong Z. Zhou Q. (2018). Dnmt3a in the dorsal dentate gyrus is a key regulator of fear renewal. *Sci. Rep.* 8 5093. 10.1038/s41598-018-23533-w 29572461 PMC5865109

[B16] Guo X. Fu Y. Zhang Y. Wang T. Lu L. Luo X. (2020). Replicated risk CACNA1C variants for major psychiatric disorders may serve as potential therapeutic targets for the shared depressive endophenotype. *J. Neurosci. Cogn. Stud.* 4 1. 34046650 PMC8153461

[B17] Hodes G. E. Pfau M. L. Purushothaman I. Ahn H. F. Golden S. A. Christoffel D. J. (2015). Sex Differences in Nucleus Accumbens Transcriptome Profiles Associated with Susceptibility versus Resilience to Subchronic Variable Stress. *J. Neurosci.* 35 16362–16376. 10.1523/JNEUROSCI.1392-15.2015 26674863 PMC4679819

[B18] Hoogland T. M. Saggau P. (2004). Facilitation of L-type Ca2+ channels in dendritic spines by activation of beta2 adrenergic receptors. *J. Neurosci.* 24 8416–8427. 10.1523/JNEUROSCI.1677-04.2004 15456814 PMC6729902

[B19] Kabir Z. D. Lee A. S. Burgdorf C. E. Fischer D. K. Rajadhyaksha A. M. Mok E. (2017). Cacna1c in the Prefrontal Cortex Regulates Depression-Related Behaviors via REDD1. *Neuropsychopharmacology* 42 2032–2042. 10.1038/npp.2016.271 27922594 PMC5561335

[B20] Kabitzke P. A. Brunner D. He D. Fazio P. A. Cox K. Sutphen J. (2018). Comprehensive analysis of two Shank3 and the Cacna1c mouse models of autism spectrum disorder. *Genes Brain Behav.* 17 4–22. 10.1111/gbb.12405 28753255

[B21] Kasim S. Egami K. Jinnah H. A. (2002). Self-biting induced by activation of L-type calcium channels in mice: serotonergic influences. *Dev. Neurosci.* 24 322–327. 10.1159/000066747 12457070

[B22] Kirac R. Eroglu L. (1991). Bay K 8644 potentiates the anxiolytic effect of ethanol. *Pharmacol. Biochem. Behav.* 39 325–327. 10.1016/0091-3057(91)90187-7 1719568

[B23] Kunchok A. Zekeridou A. McKeon A. (2019). Autoimmune glial fibrillary acidic protein astrocytopathy. *Curr. Opin. Neurol.* 32 452–458. 30724768 10.1097/WCO.0000000000000676 PMC6522205

[B24] Lai K. O. Ip N. Y. (2013). Structural plasticity of dendritic spines: the underlying mechanisms and its dysregulation in brain disorders. *Biochim. Biophys. Acta* 1832 2257–2263. 24012719 10.1016/j.bbadis.2013.08.012

[B25] LaPlant Q. Vialou V. Covington H. E. III Dumitriu D. Feng J. Warren B. L. (2010). Dnmt3a regulates emotional behavior and spine plasticity in the nucleus accumbens. *Nat. Neurosci.* 13 1137–1143. 10.1038/nn.2619 20729844 PMC2928863

[B26] Liu C. Jiao C. Wang K. Yuan N. Methylation D. (2018). and Psychiatric Disorders. *Prog. Mol. Biol. Transl. Sci.* 157 175–232. 29933950 10.1016/bs.pmbts.2018.01.006

[B27] Matt S. M. Zimmerman J. D. Lawson M. A. Bustamante A. C. Uddin M. Johnson R. W. (2018). Inhibition of DNA methylation with zebularine alters lipopolysaccharide-induced sickness behavior and neuroinflammation in mice. *Front. Neurosci.* 12:636. 10.3389/fnins.2018.00636 30279646 PMC6153314

[B28] Miousse I. R. Pathak R. Garg S. Skinner C. M. Melnyk S. Pavliv O. (2017). Short-term dietary methionine supplementation affects one-carbon metabolism and DNA methylation in the mouse gut and leads to altered microbiome profiles, barrier function, gene expression and histomorphology. *Genes Nutr.* 12 22. 10.1186/s12263-017-0576-0 28904640 PMC5588631

[B29] Mogilnicka E. Czyrak A. Maj J. (1988). BAY K 8644 enhances immobility in the mouse behavioral despair test, an effect blocked by nifedipine. *Eur. J. Pharmacol.* 151 307–311. 10.1016/0014-2999(88)90813-8 2458947

[B30] Moskvina V. Craddock N. Holmans P. Nikolov I. Pahwa J. S. Green E. (2009). Gene-wide analyses of genome-wide association data sets: evidence for multiple common risk alleles for schizophrenia and bipolar disorder and for overlap in genetic risk. *Mol. Psychiatry* 14 252–260. 10.1038/mp.2008.133 19065143 PMC3970088

[B31] Nugent B. M. Wright C. L. Shetty A. C. Hodes G. E. Lenz K. M. Mahurkar A. (2015). Brain feminization requires active repression of masculinization via DNA methylation. *Nat. Neurosci.* 18 690–697. 25821913 10.1038/nn.3988 PMC4519828

[B32] Olson P. A. Tkatch T. Hernandez-Lopez S. Ulrich S. Ilijic E. Mugnaini E. (2005). G-protein-coupled receptor modulation of striatal CaV1.3 L-type Ca2+ channels is dependent on a Shank-binding domain. *J. Neurosci.* 25 1050–1062. 10.1523/JNEUROSCI.3327-04.2005 15689540 PMC6725968

[B33] Qiao H. Li M. X. Xu C. Chen H. B. An S. C. Ma X. M. (2016). Dendritic Spines in Depression: What We Learned from Animal Models. *Neural Plast.* 2016 8056370. 10.1155/2016/8056370 PMC4736982 26881133

[B34] Ramos-Lopez O. Arpon A. Riezu-Boj J. I. Milagro F. I. Mansego M. L. Martinez J. A. (2018). DNA methylation patterns at sweet taste transducing genes are associated with BMI and carbohydrate intake in an adult population. *Appetite* 120 230–239. 10.1016/j.appet.2017.09.004 28888730

[B35] Rauch P. J. Gopakumar J. Silver A. J. Nachun D. Ahmad H. McConkey M. (2023). Loss-of-function mutations in Dnmt3a and Tet2 lead to accelerated atherosclerosis and concordant macrophage phenotypes. *Nat. Cardiovasc. Res.* 2 805–818. 39196062 10.1038/s44161-023-00326-7

[B36] Sales A. J. Joca S. R. L. (2018). Antidepressant administration modulates stress-induced DNA methylation and DNA methyltransferase expression in rat prefrontal cortex and hippocampus. *Behav. Brain Res.* 343 8–15. 29378290 10.1016/j.bbr.2018.01.022

[B37] Sales A. J. Maciel I. S. Suavinha A. Joca S. R. L. (2021). Modulation of DNA methylation and gene expression in rodent cortical neuroplasticity pathways exerts rapid antidepressant-like effects. *Mol. Neurobiol.* 58 777–794. 10.1007/s12035-020-02145-4 33025509

[B38] Shelton R. C. Grebb J. A. Freed W. J. (1987). Induction of seizures in mice by intracerebroventricular administration of the calcium channel agonist BAY k 8644. *Brain Res.* 402 399–402. 10.1016/0006-8993(87)90054-0 2435367

[B39] Sinnegger-Brauns M. J. Huber I. G. Koschak A. Wild C. Obermair G. J. Einzinger U. (2009). Expression and 1,4-dihydropyridine-binding properties of brain L-type calcium channel isoforms. *Mol. Pharmacol.* 75 407–414. 10.1124/mol.108.049981 19029287

[B40] Smedler E. Louhivuori L. Romanov R. A. Masini D. Dehnisch Ellstrom I. Wang C. (2022). Disrupted Cacna1c gene expression perturbs spontaneous Ca(2+) activity causing abnormal brain development and increased anxiety. *Proc. Natl. Acad. Sci. U. S. A.* 119 7. 10.1073/pnas.2108768119 35135875 PMC8851547

[B41] Wang X. Jiang L. Ma W. Zheng X. He E. Zhang B. (2022). Maternal separation affects anxiety-like behavior beginning in adolescence and continuing through adulthood and related to Dnmt3a expression. *J. Neurophysiol.* 128 611–618. 10.1152/jn.00247.2022 35946792

[B42] Whiteford H. A. Degenhardt L. Rehm J. Baxter A. J. Ferrari A. J. Erskine H. E. (2013). Global burden of disease attributable to mental and substance use disorders: findings from the Global Burden of Disease Study 2010. *Lancet* 382 1575–1586. 23993280 10.1016/S0140-6736(13)61611-6

[B43] Yue C. Luan W. Gu H. Qiu D. Ding X. Liu P. (2023). The role of the gut-microbiota-brain axis via the subdiaphragmatic vagus nerve in chronic inflammatory pain and comorbid spatial working memory impairment in complete Freund’s adjuvant mice. *J. Psychiatr Res.* 166 61–73. 10.1016/j.jpsychires.2023.09.003 37741061

[B44] Zhao M. Xu T. Lei J. Ji B. Gao Q. (2022). Unveiling the Role of DNA Methylation in Vascular CACNA1C Tissue-Specific Expression. *Front. Cardiovasc. Med.* 9:872977. 10.3389/fcvm.2022.872977 35711357 PMC9197502

[B45] Zheng J. Zhao Q. Ma Y. Tian J. Sun L. Zhang Z. (2023). Agomelatine enhances the therapeutic effect of venlafaxine on depression and improves the levels of S100B and GFAP. *Am. J. Transl. Res.* 15 5528–5535. 37692959 PMC10492076

[B46] Zheng Z. Guo C. Li M. Yang L. Liu P. Zhang X. (2022). Hypothalamus-habenula potentiation encodes chronic stress experience and drives depression onset. *Neuron* 110 1400–1415. 10.1016/j.neuron.2022.01.011 35114101

